# A protein elicitor PeVn1 from *Verticillium nonalfalfae* HW recognized as a MAMP triggers plant immunity response

**DOI:** 10.3389/fpls.2024.1468437

**Published:** 2024-10-10

**Authors:** Ziyu Zhang, Dong Wang, Baozhu Dong, Yu Wang, Jialu Xu, Jianxiu Hao, Hongyou Zhou

**Affiliations:** College of Horticulture and Plant Protection, Inner Mongolia Agricultural University, Key Laboratory of Biopesticide Creation and Resource Utilization for Autonomous Region Higher Education Institutions, Hohhot, China

**Keywords:** elicitors, *Verticillium nonalfalfae*, PeVn1, resistance, microbe-associated molecular pattern

## Abstract

Protein elicitors can induce plant systemic resistance to pathogens. The recognition of a potential elicitor activates intracellular signaling events, leading to plant resistance against pathogens. In this study, a novel protein elicitor was isolated from the culture filtrate of *Verticillium nonalfalfae* and named PeVn1, which can induce cell death in several plant species. The PeVn1 gene was then cloned and expressed in *Escherichia coli*. The recombinant protein PeVn1 triggers cell death in *Nicotiana benthamiana* in *NbBAK1* and *NbSOBIR1* dependent manner. Through bioassay analysis showed that the recombinant PeVn1 induced early defense induction events, such as reactive oxygen species burst, callose deposition and the activation of defense hormone signaling pathways and defense enzyme activities. Moreover, PeVn1 significantly enhanced resistance of *Nicotiana benthamiana* to *Sclerotinia sclerotiorum*, *Botrytis cinerea* and *N. benthamiana* mosaic virus and tomato to *Pseudomonas syringae* pv. *Tomato* DC3000. In conclusion, our study reveals that PeVn1 protein as a microbe-associated molecular pattern can induce plant immune responses, which provides a theoretical basis for the development of novel protein-induced disease resistance agents.

## 1 Introduction

Plant immune system is divided into two main layers, the first basic immune response is triggered mainly by pattern recognition receptors (PRRs) located on the surface of plant cells that recognize highly conserved Microbe-Associated Molecular Patterns (MAMPs) from pathogenic or non-pathogenic bacteria (Boller and He, 2009)), which are essential for microbial growth or lifestyles (Zipfel and Felix, 2005; Jones and Dangl, 2006). Thus the defense response induced by PRRs mediated perception of MAMP is also known as Pattern-Triggered Immunity (PTI) or Microbe-Associated Molecular Patterns-Triggered Immunity MAMP-Triggered Immunity (MTI) (Liu et al., 2014). This response is accompanied by disease-fighting responses such as rapid changes in intracellular ion levels, bursts of reactive oxygen species (ROS), calcium-dependent protein kinases (CDPK), activation of Mitogen-Activated Protein Kinases (MAPK) cascades, activation of the expression of a number of related immune genes, and callosal deposition that can effectively inhibit the invasion of the vast majority of pathogenic bacteria in the environment (Boudsocq and Sheen, 2013; Ranf et al., 2015). Many PRRs rely on BRASSINOSTEROID INSENSITIVE 1-ASSOCIATED RECEPTOR KINASE 1 (BAK1) family proteins as well as SUPPRESSOR OF BIR1-1 (SOBIR1) to trigger defense responses (Liebrand et al., 2014). ROS are believed to be a signaling molecule for plant defense and interact with other signaling networks in plants (Miller et al., 2008). ROS accumulation is controlled by enzymes that detoxify ROS, such as superoxide dismutase (SOD), catalase (CAT), ascorbate peroxidase (POD), and glutathione reductase (Apel and Hirt, 2004).

To date, a large number of MAMP/PAMP have been reported in plant pathogenic viruses, bacteria, fungi and oomycetes, and these MAMP can be categorized according to the type of substances: proteins, organic glycoconjugates, lipids or lipopeptides (Zipfel and Felix, 2005; Boudsocq and Sheen, 2013). In bacteria flagellin is one of the most studied MAMPs, and the Arabidopsis receptor FLS2, which recognizes bacterial flagellin, belongs to the LRR-RLK. A conserved 22-amino-acid sequence at the N-terminus of bacterial flagellin (flg22) is sufficient for FLS2 recognition (Zipfel and Felix, 2005). In fungi chitin is one of the most studied MAMPs. Chitin is recognized by the phytochitin receptor (CERK1) and lysine motif receptor kinase 5 (LYK5), mediating downstream immune responses (Langner and Göhre, 2016). In addition, proteins secreted by pathogenic bacteria, also known as MAMPs, are inducing an immune response and enhancing plant resistance by referring to classes of MAMPs as elicitor, which can be categorized as proteins, glycans, peptides, lipids, and others (Boutrot and Zipfel, 2017).

Elicitors induced defense responses are currently receiving a great deal of attention in the study of plant induced resistance responses (Czobor et al., 2017). The protein elicitor PeBL1 was previously screened from *Brevibacillus laterosporus* strain A60 (Wang et al., 2015). This elicitor stimulates typical defense responses, such as allergic responses, ROS bursts, extracellular alkalinization, and lignin accumulation in *N. benthamiana* leaves and increases *N. benthamiana* resistance to tobacco mosaic virus (TMV)-GFP and systemic resistance to *Pseudomonas syringae* pv. *tabaci*. PevD1 from *Verticillium dahliae* induces the accumulation of phenolics, callose, and lignin and enhances resistance to TMV and *V. dahliae* infection in *N. benthamiana* (Wang et al., 2012; Liang et al., 2018). The protein elicitor PeSy1 isolated from strain *Sclerotinia yanglingensis* Hhs.015 induced resistance to *N. benthamiana* and acted as a Microbial-Associated Molecular Patterns (MAMP) to induce an immune response in the plant, which enhanced resistance to *S. sclerotiorum*, *Phytophthora capsica* and other pathogenic bacteria (Wang et al., 2023). Although numerous elicitors have been identified, our comprehension of how these agents initiate plant defense responses remains incomplete (Pršić and Ongena, 2020).

*Verticillium nonalfalfae* is another pathogenic species that has a more limited host range than *V. dahliae* (Inderbitzin et al., 2011). However, it is a causal agent of *Verticillium* wilt, which results in the death of several important crops (Inderbitzin and Subbarao, 2014). The infestation of hops (Humulus lupulus L.) by *V. nonalfalfae* has been reported with the occurrence of strains of different virulence. Pathogenic strains can lead to a reduction in hops production. By analyzing the whole genome sequencing of *V. nonalfalfae*, a large number of secreted proteins have been obtained, among which key proteins have been identified as virulence factors (Marton et al., 2018). The endoglucanase VdEG-1 (Novo et al., 2006) and the specific secreted protein VdSSP1 (Liu et al., 2013) are further potential carbohydrate-degrading enzymes important for *V. dahliae* pathogenicity. VnaPRX1.1277 and VnaSSP4.2 are required virulence factors to promote colonization of hop plants by *V. dahliae* (Flajsman et al., 2016). The secreted Aspf2-like protein VDAL in *V. dahliae* is responsible for the wilting of plant leaves. Overexpression of VDAL in *Arabidopsis thaliana* and cotton resulted in enhanced resistance of the overexpressed plants to *V. dahliae*, while not affecting normal plant growth and development (Jiang et al., 2022). Ave1 is a secreted protein that is specific to the *V. dahliae* race 1. It is capable of triggering immunity by recognizing the Ve1 tomato resistance receptor (Jonge et al., 2012). Consequently, elicitors that activate plant immunity can be used to enhance plant disease resistance.

Nevertheless, many *V. nonalfalfae* secreted proteins regulating plant immunity remain to be characterized. In this study, a novel secreted MAMP, named PeVn1, was isolated and characterized from *V. nonalfalfae*. PeVn1 stimulates disease resistance and induces immune responses in *N. benthamiana*. Moreover, it is dependent on *BAK1* and *SOBIR1* to induce plant cell death, further elucidating the mechanisms by which PeVn1 induces plant immunity. Our findings provide a potential strategy for the biological control of agricultural diseases.

## 2 Results

### 2.1 Isolation and identification of the *V. nonalfalfae* HW protein MAMP

**Figure 1:**
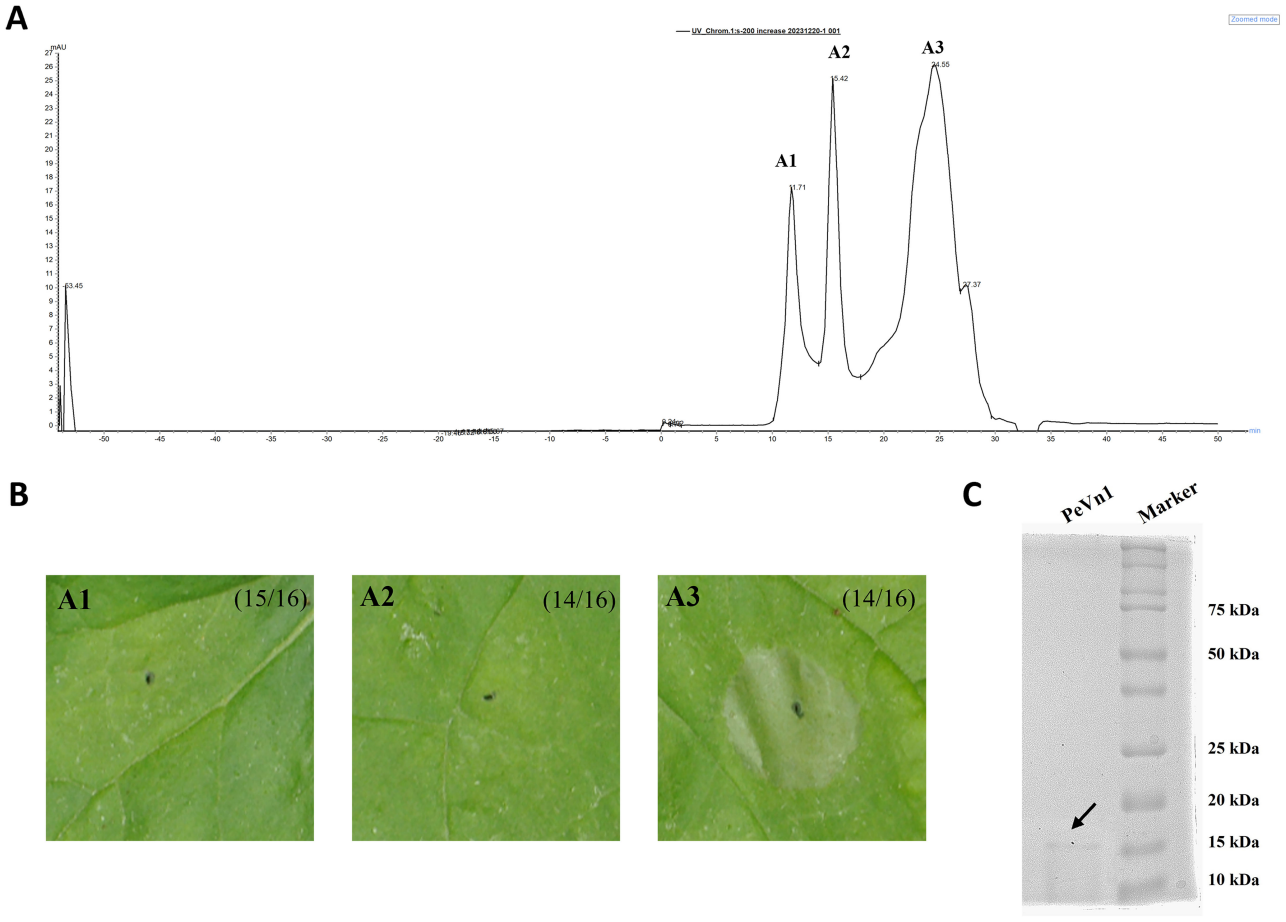
Separation and purification of MAMP with potential cell death activity from *V. nonalfalfae* HW. **(A)** Chromatogram by Superdex 200 increase 10/300 GL gel column. **(B)** Cell death activity was determined by pairwise fractionation of fractions 1, 2 and 3 obtained from fractions 1, 2 and 3. **(C)** Detection of fraction 3 by Thomas blue staining, SDS-PAGE running gel. black arrows represent the target proteins of fraction 3.

This study showed that the supernatant from the culture filtrate broth of *V. nonalfalfae* HW induced cell death in *N. benthamiana* leaves. Crude proteins were extracted from the culture filtrate supernatant at a saturated concentration of 75% ammonium sulfate and dialyzed to induce cell death in the *N. benthamiana* leaves (**Supplementary Figures 1**). The active fractions were purified using a Superdex 200 increase 10/300 GL, yielding three peaks (**Figure 1A**).The fraction corresponding to peak A3 induced cell death activity in tobacco (**Figure 1B**). As shown in SDS-PAGE, a single band was identified from A3 with a size of about 15 kDa. To determine the identity of the protein present in A3, a single SDS-PAGE band was cut and identified by mass spectrometry (**Figure 1C**). Based on liquid chromatography tandem mass spectrometry, reliable sequences were obtained for six peptides: (a) SMAVASTTWTIENTR, (b) QIVWPAYTDKQLAK, (c) AVVVKPNQNYPVQALP, (d) QLAKAVVVKPNQNYPVQALP, (e) SSKQIVWPAYTDK and (f) ANDCTWTFSVNTGSSNTPCTFHTK. The peptides showed high similarity to the putative protein from *V. nonalfalfae* VnAa140/NRRL 66861 by sequence comparison using BLASTp (XP. 028497723.1). Based on these results, the coding sequence of the protein secreted from *V. nonalfalfae* HW was determined. The coding sequence was cloned into a *pET* vector (with a His tag) with the signal peptide region removed and expressed in *E. coli* BL21 cells. The recombinant protein was purified using HisPur Ni-NTA resin. Its molecular weight was approximately 15 kDa according to SDS-PAGE (**Supplementary Figures 2A**). Cell death was detected by infiltrated *N. benthamiana* leaves in the recombinant protein. The results demonstrated that the recombinant protein could induce cell death after 3 days (**Supplementary Figures 2B**). Leaf cell death was observed by trypan blue staining. We hypothesized that PeVn1 is a MAMP secreted by *V. nonalfalfae* HW.

### 2.2 PeVn1 characterization

**Figure 2:**
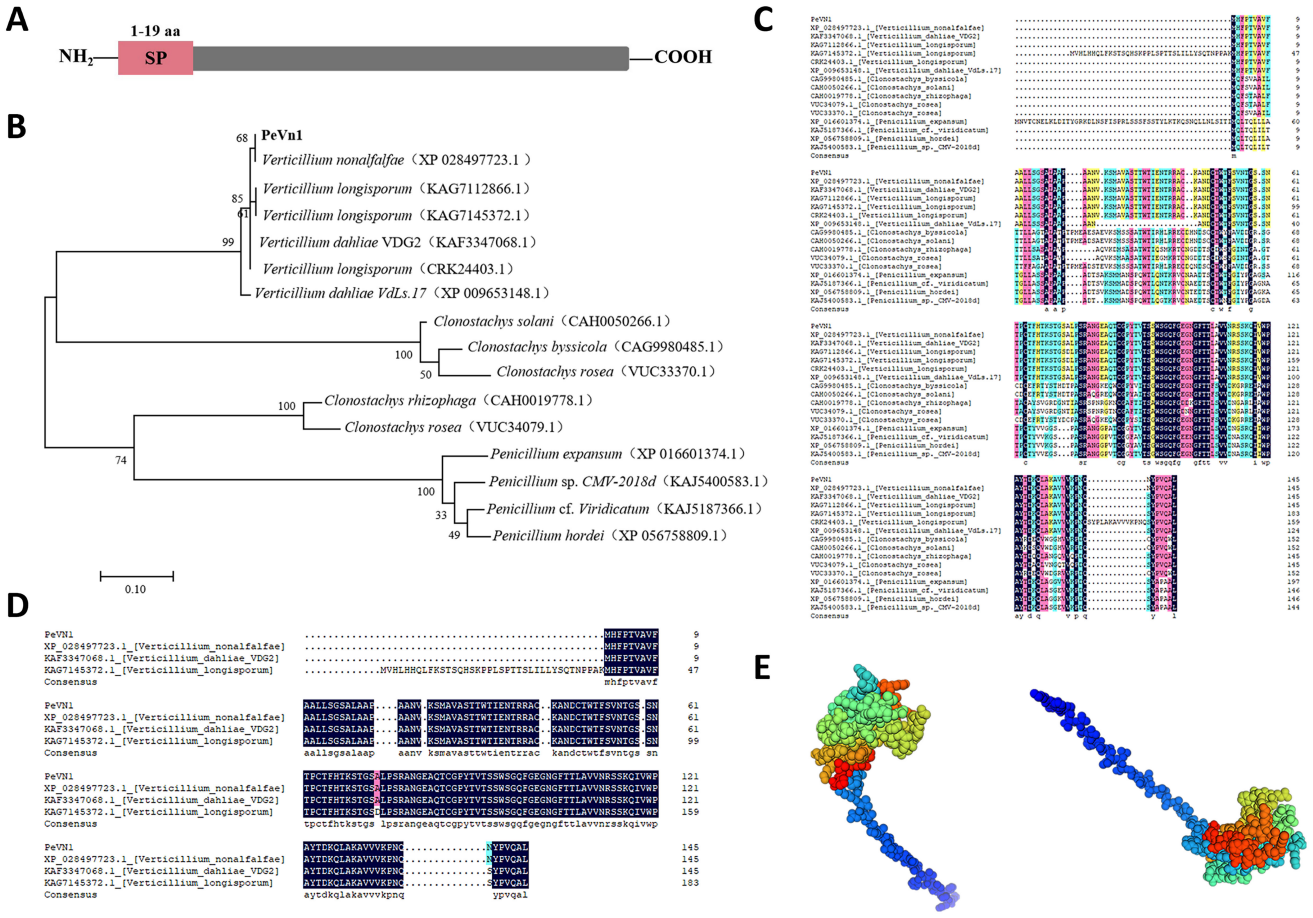
Bioinformatics analysis of PeVn1. **(A)** Signal peptide prediction of PeVn1,N-terminal 1-19 aa is the signal peptide region. **(B)** Construction of a phylogenetic tree of PeVn1 based on amino acid sequences. **(C, D)** Conserved region analysis of PeVn1. Colored boxes represent conserved amino acid. **(E)** Prediction of the tertiary structure of PeVn1.

The SMART analysis indicates that PeVn1 is a putative protein with no significant structural matches, with the exception of the signal peptide (**Figure 2A**). To further verify whether PeVn1 is a secreted protein, The PeVn1 signal peptide was tested using an invertase enzyme to examine its function for secretion in yeast (Jacobs et al., 1997). The signal peptide was inserted into the *pSUC2* vector, which was transformed into the YTK12 yeast strain. All transformants containing the positive control Avr1b signal peptide or the test PeVn1 grew well on the CMD−W and YPRAA plates, indicating that the PeVn1 signal peptide had a secretion function. In addition, the enzyme activity of the secreted invertase from the transformants was detected *in vivo*. 2,3,5-triphenyl tetrazolium chloride was converted into the insoluble red-colored 1,3,5-triphenyl formazan after adding yeast containing Avr1b or PeVn1^sp^, indicating that invertase was secreted from the yeast in the presence of the PeVn1 signal peptide (**Supplementary Figure 3**). PeVn1 consists of 146 amino acid residues, of which the first 19 are N-terminal signal peptides with a molecular weight of 15 kDa and a PI of 9.42. A phylogenetic tree was constructed using fifteen sequences with high homology to PeVn1 obtained from the NCBI database (**Figure 2B**). Multiple sequence comparisons based on homologous sequences showed that PeVn1 belongs to *Verticillium* spp., but its protein sequence was 66.32% similar (**Figure 2C**) and conserved in *Verticillium* sp., with 49.57% similarity (**Figure 2D**). The protein structure was predicted by SWISS-MODEL and the structural confidence of the best-matched structure of PeVn1 was 95.14% with only 10 β-folding (**Figure 2E**). These results suggested that PeVn1 is a secreted protein that is conserved in *V. nonalfalfae*.

### 2.3 Protein PeVn1 induces cell death in plants

**Figure 3:**
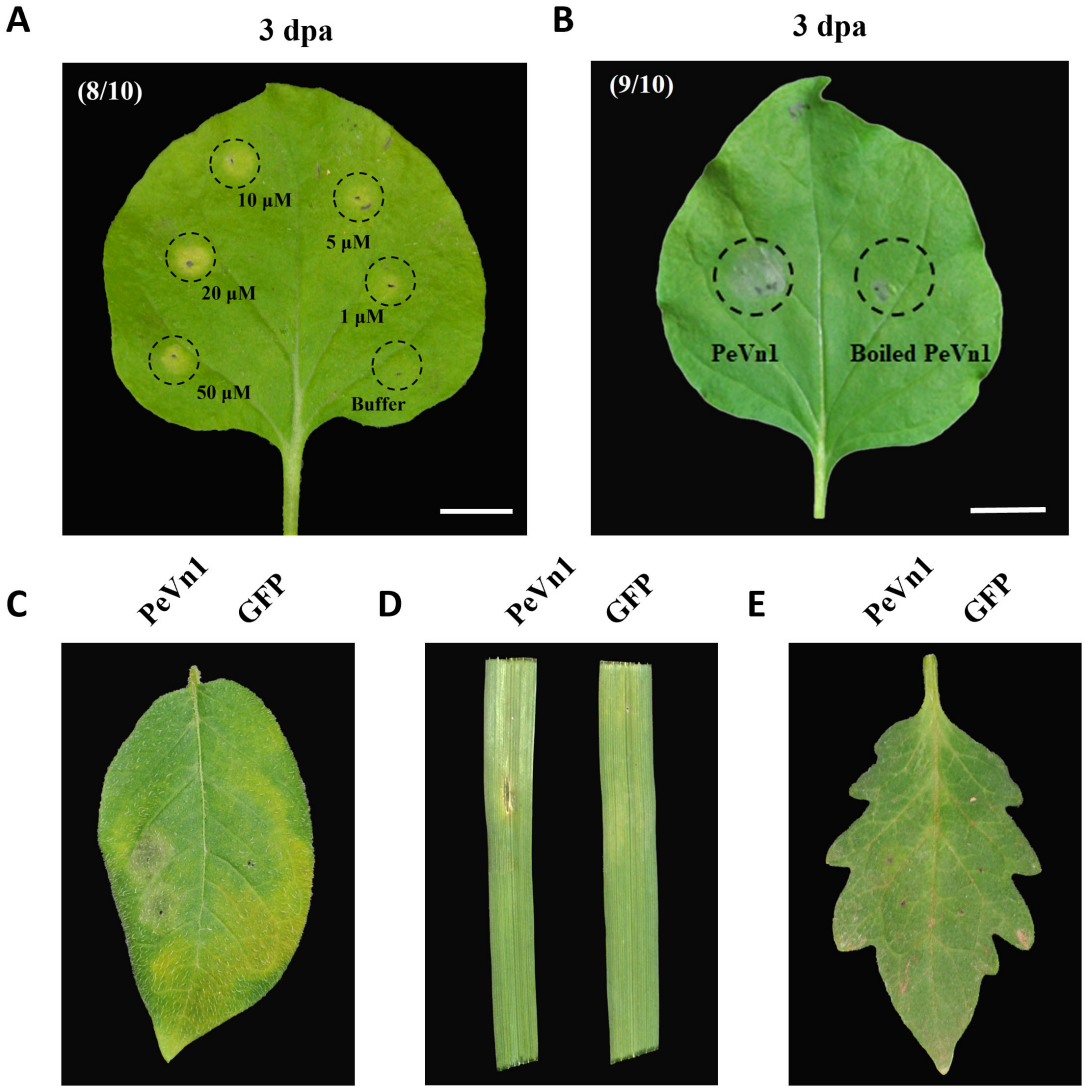
Cell death activity of PeVn1 induced in *N. benthamiana* and other plants. **(A)** Recombinant protein PeVn1 was divided into 1-50 μM and immersed into *N. benthamiana* and the HR response was observed and photographed after 3 d. **(B)** The thermal stability of PeVn1 was determined by immersing 5 μM of the recombinant protein PeVn1 in *N. benthamiana* leaves at 100°C for 15 min. **(C–E)** The detection of cell death responses induced by 5 μm PeVn1 on potato **(C)**, oat **(D)** and tomato **(E)**, 20 mM Tris-HCl as control.

The cell death activity of recombinant PeVn1 in tobacco was further analyzed. Necrotic areas appeared in *N. benthamiana* leaf blades after infiltrated in different concentrations of PeVn1, 3 days after treatment (**Figure 3A**). Cell death increased with increasing concentration, and the recombinant protein did not induce cell death at 100°C in the thermal stability test. These findings suggest that PeVn1 exhibits hypersensitive response activity in *N. benthamiana* (**Figure 3B**). In addition, we conducted cell death assays in different crops and found that PeVn1 induced cell death in potato and oat plants, while no necrotic response was observed in tomato plants, suggesting that the necrotic response induced by PeVn1 was species specific (**Figure 3C**).

### 2.4 Analysis of PeVn1 subcellular localization and signal peptide function

**Figure 4:**
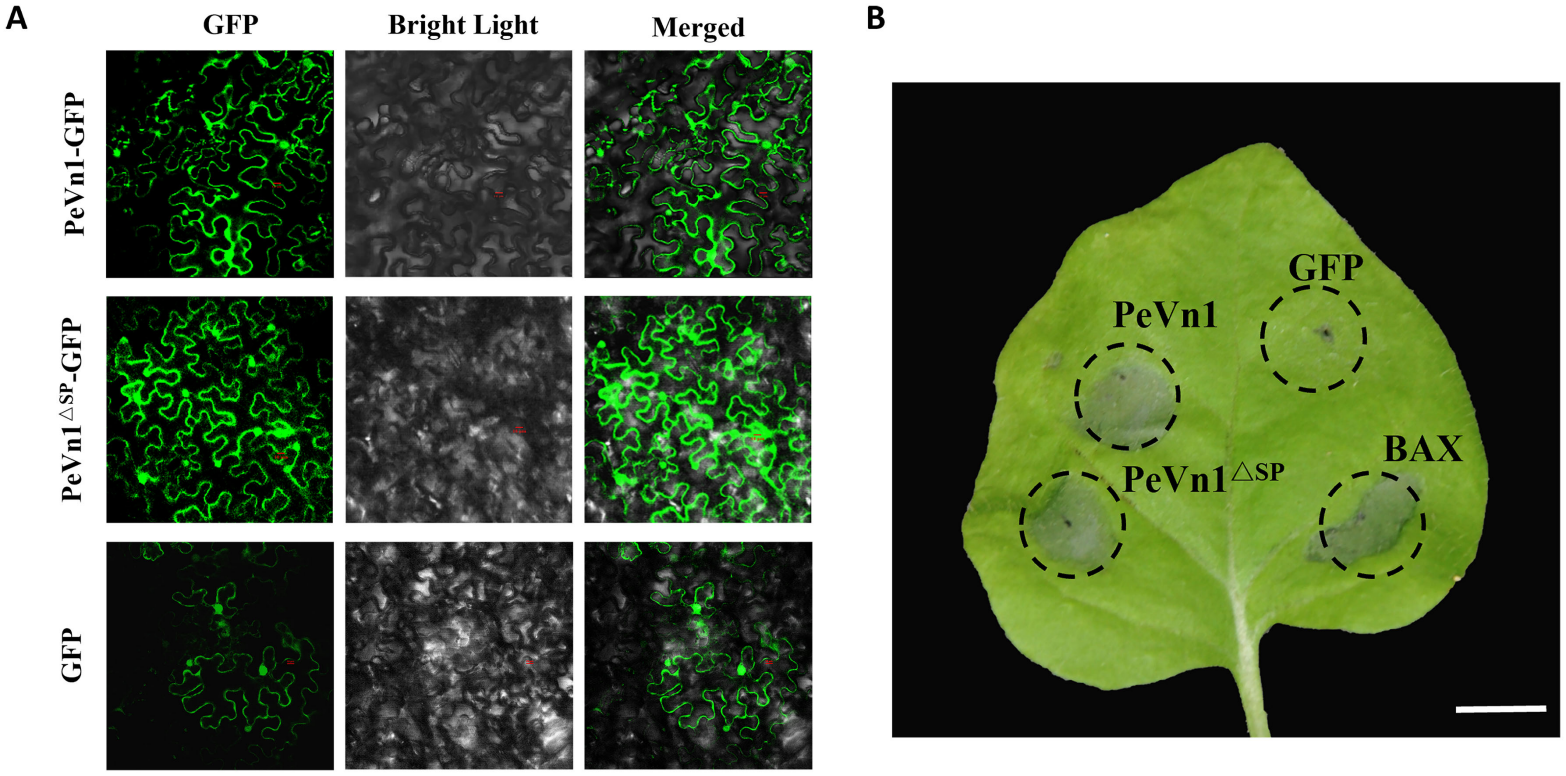
Subcellular localization and signaling peptides for PeVn1-primed immunity. **(A)** Subcellular localization of PeVn1 in *N. benthamiana* leaves. **(B)** Photographs of *N. benthamiana* leaves were taken at 4 days post infiltration. Bars are 20 µm.

To clarify the specific locations at which PeVn1 functions, its subcellular localization was analyzed. Using *N. benthamiana* leaf cells, PeVn1 was found to be localized in the plasma membrane and nucleus, as was PeVn1^ΔSP^, suggesting that the signal peptide of PeVn1 may have secretory activity (**Figure 4A**). To verify whether the inhibitory activity of PeVn1 is dependent on the signal peptide, PeVn1^ΔSP^ was constructed into an expression vector for transient expression in tobacco. The results showed that PeVn1 without the signal peptide still induced cell death in *N. benthamiana* leaves, so the stimulation of immune responses by PeVn1 may not be dependent on the signal peptide (**Figure 4B**).

### 2.5 PeVn1 triggers defense responses in *N. benthamiana*

**Figure 5:**
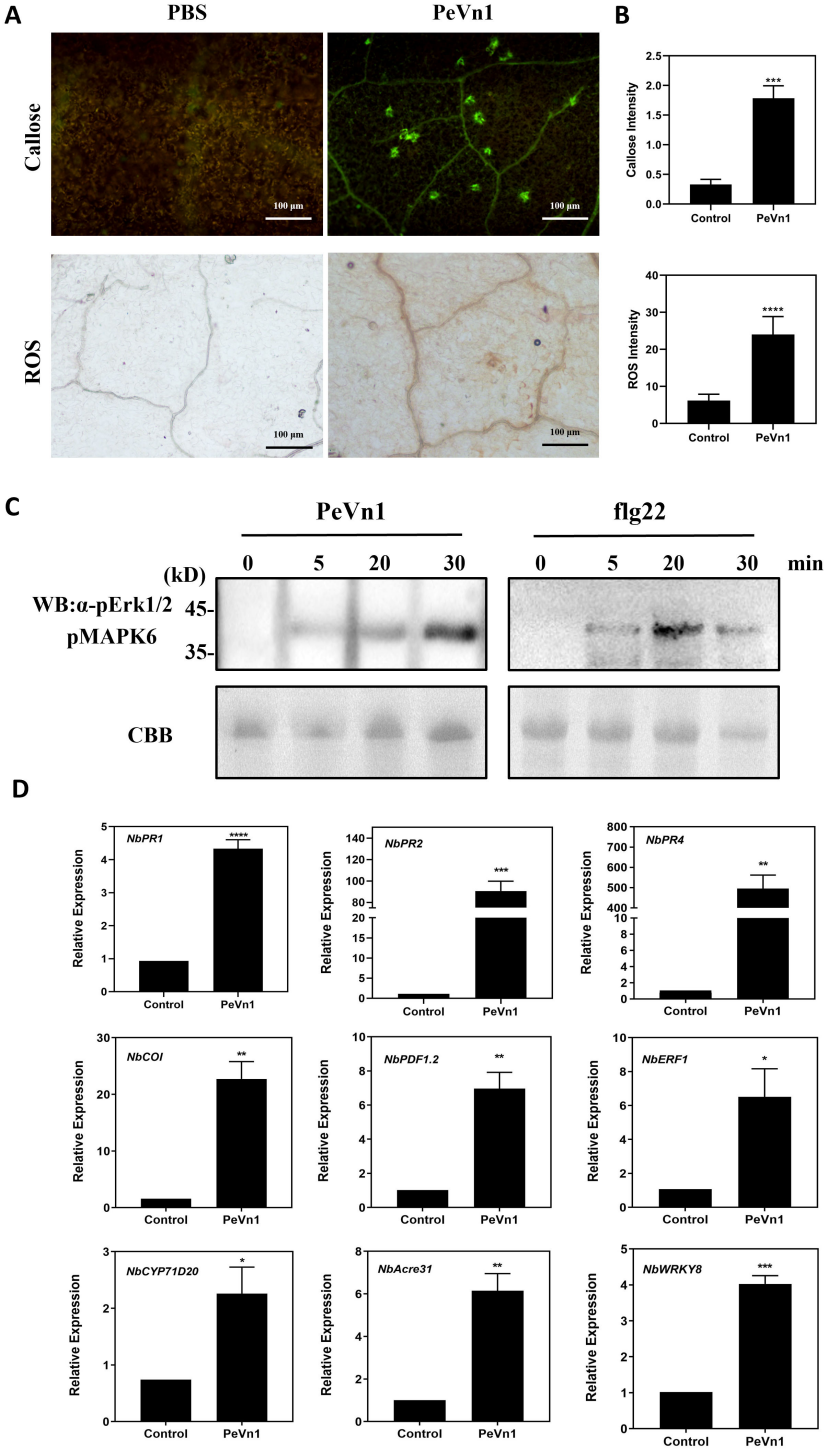
PeVn1 induces early immune responses in *N. benthamiana*. **(A)** *N. benthamiana* leaf blades were treated with 5 μM PeVn1 or buffer control for 24h, Reactive oxygen species (ROS) were observed as brick red staining after 3,3’ - diaminobenzidine (DAB) reaction (bars are 1 cm) and callose was observed as pale green fluorescence under UV light after aniline blue reaction (bars are 100 μm).**(B)** ROS and callus deposition in *N. benthamiana* leaves were quantified by the software ImageJ. Mean ± SE. **(C)** PeVn1 and flg22 induced MAPK activation in *N. benthamiana*. The leaves were infiltrated with 5 μM PeVn1 or flg22, and total proteins were extracted for immunoblotting with an anti-pERK1/2 antibody. Protein loading is indicated by Coomassie brilliant blue (CBB) staining. **(D)** *N. benthamiana* leaves were treated with 5µM recombinant protein PeVn1 and the expression of defense and PTI related genes measured after 48h. *NbEF1α* was used as an internal control gene for normalization. Mean and SE were calculated from three biological replicates. The statistical analyses were performed with Student’s t test. Bars indicate ± SE. *p < 0.1, **p < 0.01, ***p < 0.001, ****p < 0.0001.

The burst of ROS is a hallmark of plant defense response signaling during plant-microbe interactions. HR is accompanied by early plant defense responses such as ROS bursts, callus deposition and defense gene expression (Schwessinger and Ronald, 2012; Han and Hwang, 2017). To investigate whether PeVn1-induced HR is associated with plant immune responses, we injected 5 µM of the recombinant protein PeVn1 and assessed ROS accumulation using 3,3’-diaminobenzidine staining and pale green fluorescence of callose deposition in leaf cells under UV excitation after aniline blue staining (**Figures 5A, B**). The MAPK pathway has an important role in cell signaling and participates in plant immune responses (Li et al., 2022). By detecting MAPK phosphorylation, as in leaves treated with flg22, PeVn1 was able to induce MAPK activation in tobacco leaves, and the degree of activation increased with time (**Figure 5C**). To determine whether PeVn1 activates plant defense responses, genes related to PTI and defense responses in *N. benthamiana* were selected for RT-qPCR analysis. These results indicated a significant increase in the relative expression of defense response and PTI-related genes. Among these, *NbPR1* and *NbPR2* are marker genes for SA, *NbPR4* and *NbERF1* are marker genes for JA/ET, and *NbCYP71D20* and *NbAcre31* are PTI-related marker genes. Thus, PeVn1 may function as a protein exciter and MAMP that induces plant defense responses mediated by SA and JA/ET dependent signaling pathways and responds to PTI related pathway genes (**Figure 5D**).

### 2.6 PeVn1 defense-related enzyme activities

**Figure 6:**
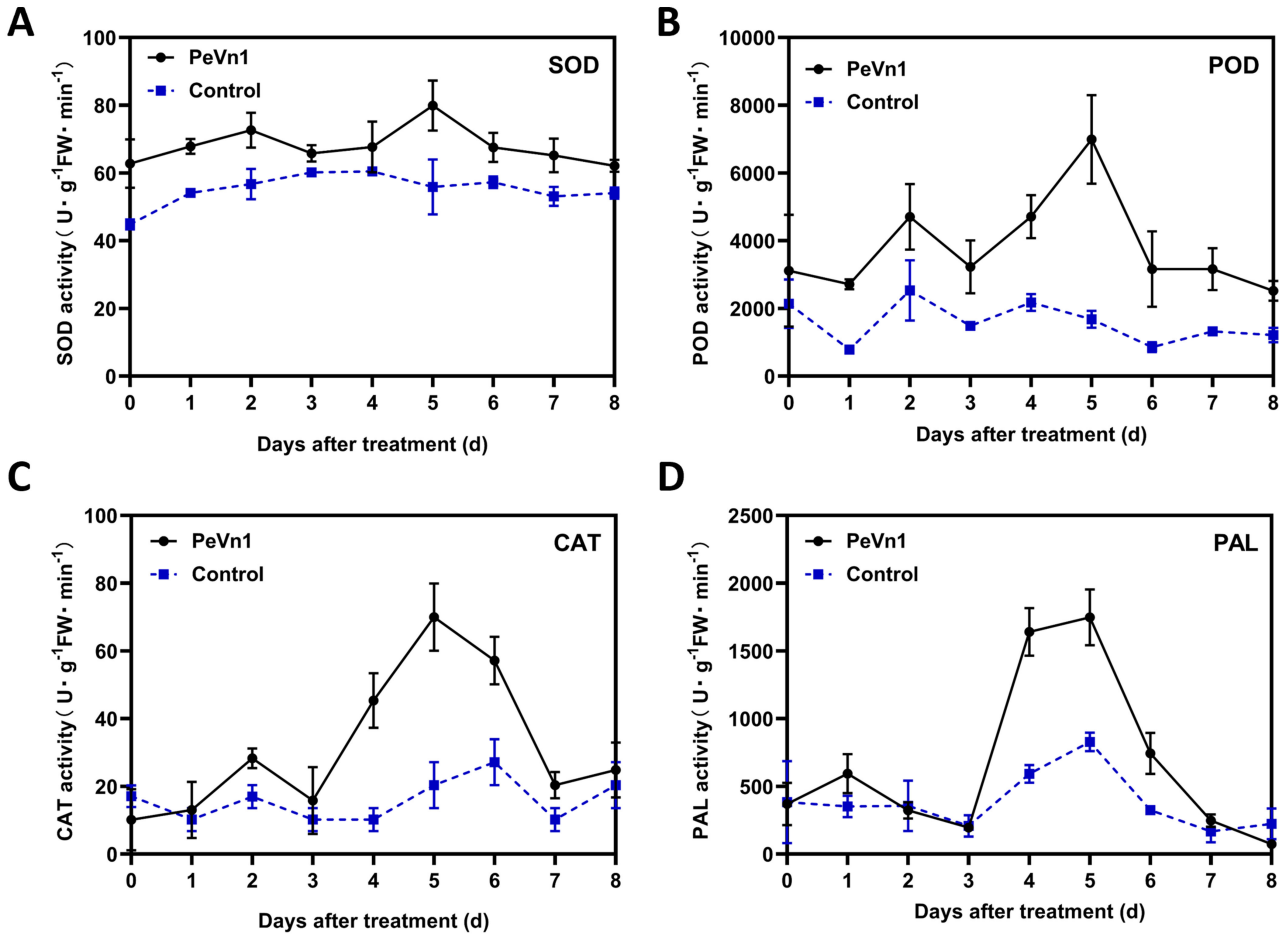
Changes in SOD **(A)**, POD **(B)**, CAT **(C)** and PAL **(D)** defense enzyme activities in PeVn1-His-treated *N. benthamiana* leaves. *N. benthamiana* leaves were treated with 5 μM PeVn1 or buffer control for 0-8 dat. Error bars indicate standard deviation among triplicates.

The accumulation of ROS is regulated by enzymes that detoxify ROS, including superoxide dismutase (SOD), peroxidase (POD), catalase (CAT), and phenylalanine ammonia-lyase (PAL) (Apel and Hirt, 2004). Enzyme activities related to defense showed a different pattern after PeVn1 treatment compared to those in the control group. Following treatment with the PeVn1 protein solution, the activities of POD, SOD, CAT and PAL gradually increased, peaking after 5 days. In general, the enzyme activity in the treated group was higher than that in the control group. The SOD activity in the treated group exhibited an initial increase on day 2, reaching a peak on day 5 with an enzyme activity of 79 U, which was 23 U higher than that in the control group. Nevertheless, this discrepancy was not statistically significant (**Figure 6A**). The peak enzyme activity of POD in the treated group was 4.25 times higher than that in the control group. Similarly, the peak CAT activity was 70 U, which was 3.4 times higher than that in the control group (**Figures 6B, C**). The peak enzyme activity of PAL was 1,747 U, which was 2.1 times higher than that of the control group (**Figure 6D**).

### 2.7 Immune responses stimulated by PeVn1 are dependent on *NbBAK1* and *NbSOBIR1*

**Figure 7:**
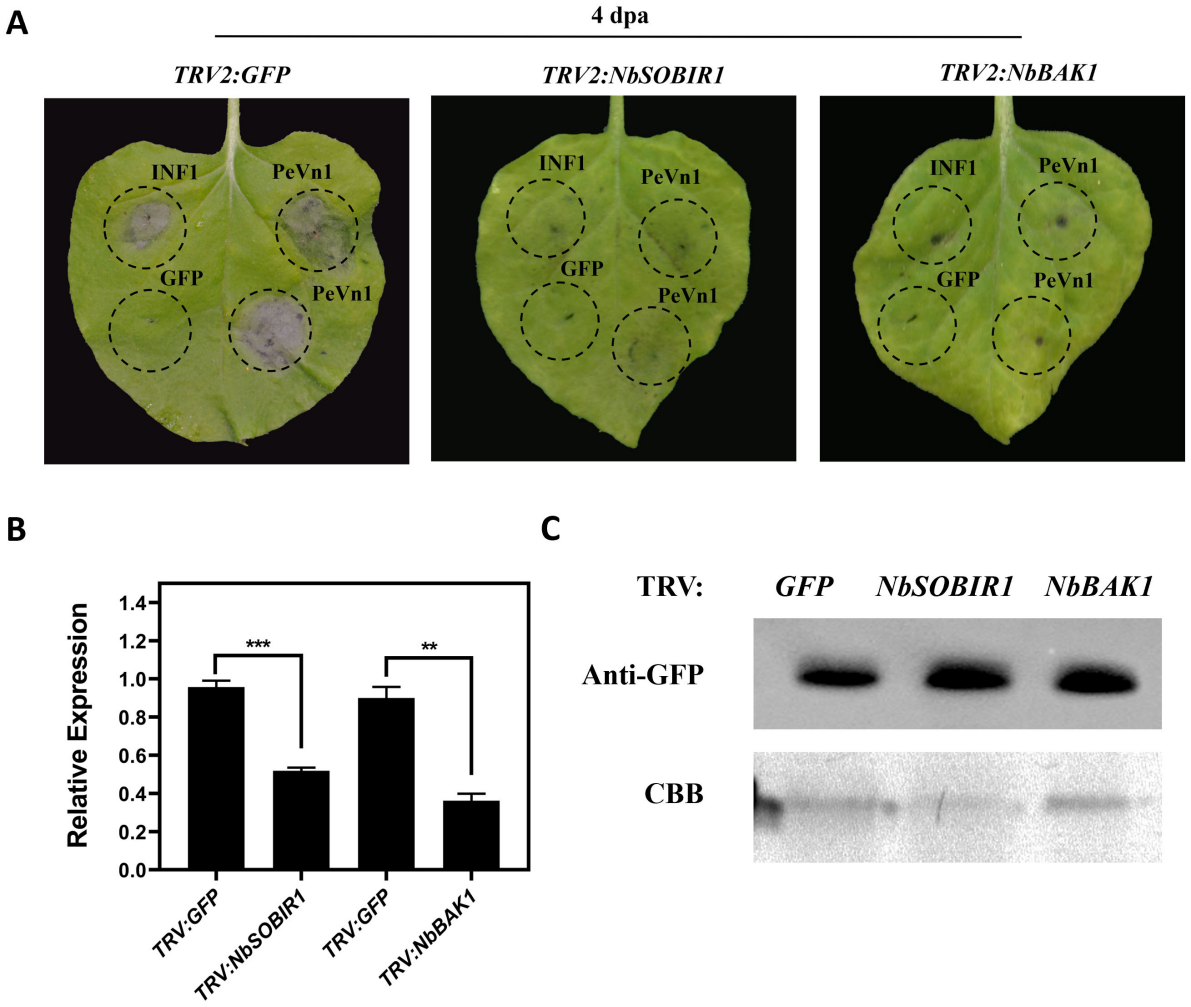
Required for PeVn1-induced cell death are *NbBAK1* and *NbSOBIR1*. **(A)** The *TRV2* construct of *Agrobacterium tumefaciens* was infiltrated into the gene-silenced plants. Photographs were taken 4 days post-infiltration (dpa), and GFP-silenced plants were used as controls. **(B)** Expression levels of *NbBAK1* and *NbSOBIR1* after VIGS treatment as assessed by quantitative reverse transcription PCR. *NbEF1α* was used as an internal reference gene. **(C)** Western blotting analysis of transiently expressed PeVn1 in *NbBAK1* and *NbSOBIR1* silenced *N. benthamiana* leaves. Protein loading is indicated by Coomassie brilliant blue (CBB) staining. *p < 0.1, **p < 0.01, ***p < 0.001.

It has been shown that *NbBAK1* and *NbSOBIR1* receptor kinases are involved in a variety of plant immune responses in plant immunity. Therefore, to test whether *NbBAK1* and *NbSOBIR1* mediate PeVn1 induced cell death in *N. benthamiana*, we used VIGS to silence *NbBAK1* and *NbSOBIR1* in *N. benthamiana* leaf blades, using TRV-GFP as the empty control and *TRV-INF1* as the positive control. PeVn1 did not induce cell death in the silenced *NbBAK1* and *NbSOBIR1* plants (**Figure 7A**). Other silenced leaves were extracted for a RT-qPCR silencing efficiency assay when albino symptoms appeared, and the silencing efficiencies of *NbBAK1* and *NbSOBIR1* were found to be between 52% and 64% (**Figure 7B**). The results showed that PeVn1 did not induce cell death in *NbBAK1* and *NbSOBIR1* silenced plants. Western blotting showed that PeVn1 was expressed in the leaves of both *NbBAK1* and *NbSOBIR1* silenced plants (**Figure 7C**). These results suggest that PeVn1 may be a MAMP whose induction of plant cell death is dependent on *NbBAK1* and *NbSOBIR1*.

### 2.8 PeVn1 enhances plant disease resistance

**Figure 8:**
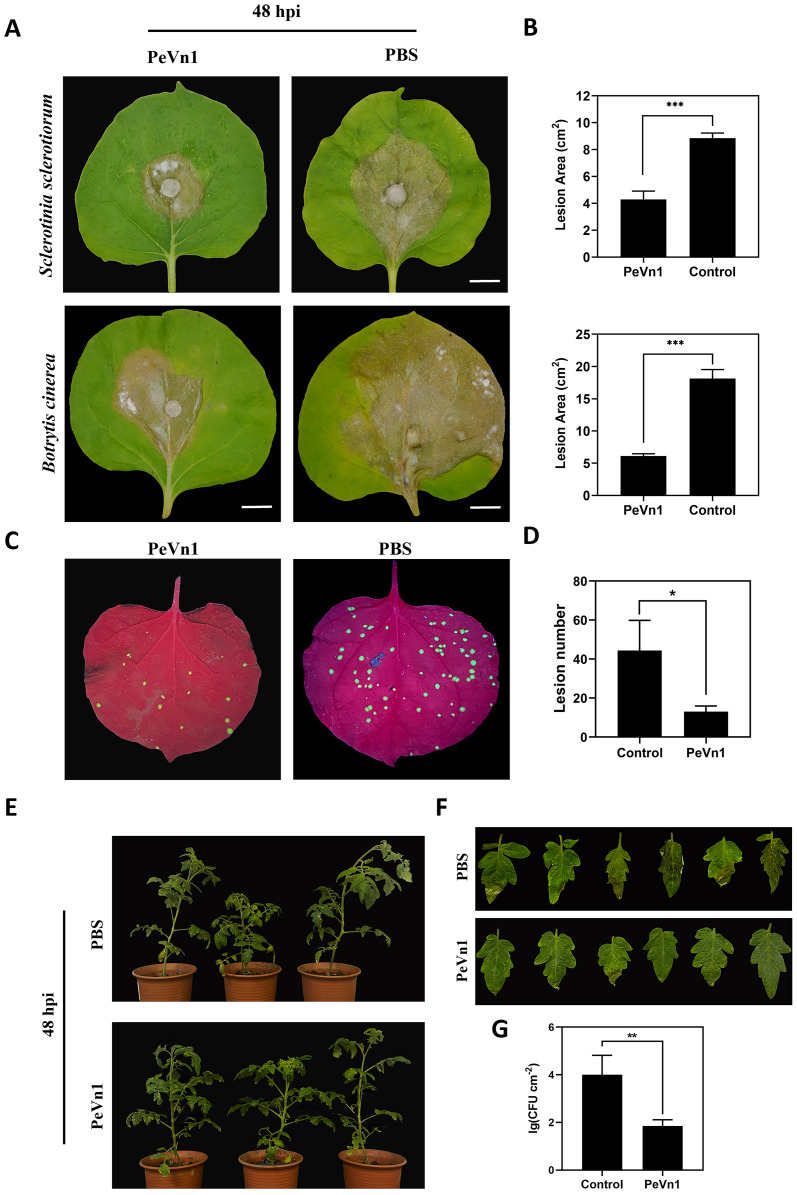
Analysis of PeVn1-induced disease resistance. **(A, B)** Representative leaves showing *Sclerotinia sclerotiorum* and *Botrytis cinerea* infection after treatment with 5 μM PeVn1 or phosphate-buffered saline (PBS) control. *N. benthamiana* leaves were inoculated with *S. sclerotiorum* and *B*. *cinerea* after PeVn1 treatment for 24 h. **(C)** 5 μM recombinant protein PeVn1 was immersed into *N. benthamiana* leaf blades and 24 h later, TMV-GFP supernatant was frictionally inoculated into *N. benthamiana* leaf blades and 4 d later, the photos were taken under the UV lamp. **(D)** Calculation of TMV in *N. benthamiana* leaf blades. *N. benthamiana* leaves; the number of TMV-GFP spots decreased significantly after PeVn1 treatment **(E–G)** Recombinant protein 5 μM PeVn1 was sprayed and inoculated onto tomato leaves and after 3 d of moist incubation, *Pst* DC3000 bacterial suspension was sprayed and inoculated onto tomato leaves and after 3 d of incubation, tomato leaves were photographed, and the number of colonies was counted. The number of colonies in tomato leaves was significantly reduced after PeVn1 treatment. The control group was PBS buffer. The experiment was conducted three times with similar results. Bars are 1 cm. Bars indicate ± SE. *p < 0.1, **p < 0.01, ***p < 0.001.

To verify whether PeVn1 can induce plant disease resistance, we inoculated *N. benthamiana* leaves with the plant pathogenic fungi *S. sclerotiorum* and *B. cinerea* and *N. benthamiana* mosaic virus green fluorescent protein (TMV-GFP). PeVn1 at a concentration of 5 μM was immersed into the leaves separately and inoculated with the pathogenic fungi 24 h later. The results showed that the lesion areas of *S. sclerotiorum* and *B. cinerea* were reduced by 53.5% and 69%, respectively, at 48 h after inoculation (**Figures 8A, B**). Similarly, the number of TMV-GFP lesions was reduced in PeVn1-treated plants compared to the control (**Figures 8C, D**), indicating that PeVn1 can inhibit the growth of plant pathogens and induce disease resistance in plants. In addition to plant leaves, tomato plants have also been used to study the resistance of PeVn1 to bacterial pathogens. Tomato leaves were sprayed with recombinant protein of PeVn1 at a concentration of 5 μM, and 24 h later, *Pst* DC3000 was inoculated into tomato plants. The results showed that the density of pathogens and spots in PeVn1-treated tomato plants were significantly reduced compared to the control (**Figures 8E–G**).The results showed that PeVn1 could inhibit the proliferation of fungi and viruses in tobacco and in bacteria in tomato.

## 3 Discussion

The investigation and development of plant immune priming mechanisms have provided novel methodologies and perspectives for plant protection. Protein elicitors not only induce the ability of plants to resist pathogenic invasion but also exhibit stress resistance and growth promoting functions. In this study, a novel protein elicitor PeVn1, was isolated and identified from the culture filtrate of *V. nonalfalfae*. The recombinant PeVn1 purified induces cell death in *N. benthamiana.* The cell death activity was enhanced with increasing concentration. In addition, PeVn1 does not cause cell death after incubation at 100°C for 15 min. Cell death can also be induced by PeVn1 in other plant species such as potato and oat (Midgley et al., 2022).The recognition of MAMP by plants induces early plant defense responses, including ROS bursts and callose deposition (Sun and Zhang, 2020). Research has shown that PeVn1 induces ROS bursts, callus deposition, and activation of the MAPK signaling pathway. In addition, PeVn1 resulted in significant upregulated of the expression of two PTI genes (*NbCYP71D20* and *NbAcre31*), and these results suggest that PeVn1 acts as a MAMP to trigger an immune response in *N. benthamiana*.

Proteins secreted by cells are typically characterized by low molecular weight and high cysteine residue content. These characteristics play a pivotal role in the cells’ crucial defense functions (Templeton et al., 1994). As an example, VmE02 comprising 146 amino acids and ten cysteine residues, initiates plant immune responses and enhances *N. benthamiana* resistance against *S. sclerotiorum* and *P. capsici* (Nie et al., 2019). The SCRP protein SsSSVP1^ΔSP^ identified in *S. sclerotiorum* can interact with the conserved cytochrome b-c1 complex subunit QCR8 in plants, thereby eliciting an immune response (Lyu et al., 2016). In our study, PeVn1 possessing a molecular weight of 14.6 kDa and four cysteine residues, shared amino acid sequence identity with the uncharacterized protein, D7B24_002174, although without an identified domain. Signal peptide prediction revealed that PeVn1 contains 19 N-terminal signal peptide residues, classifying it as a canonical secretory protein (**Figure 2A**).

The plant plastid exosome space is an important site for pathogen invasion, and plants and pathogens have a close relationship (Orozco-Cárdenas et al., 2001; Garcia-Brugger et al., 2006). To clarify the specific location where PeVn1 exerts its function, transient expression and subcellular localization of PeVn1 were analyzed. We found that PeVn1 induced cell death with or without SP (**Figure 4**). The subcellular localization of PeVn1 is localized to the plasma membrane and nucleus, suggesting that it has multiple pathways of action. Plants use PRRs on cell membranes to recognize PAMPs (polysaccharide substances of fungi, bacterial flagellin, and Harpin proteins) to elicit PTIs, which are widely distributed and conserved in plant pathogenic bacteria. Plants contain two types of PRRs, including Receptor-Like Proteins (RLPs), which contain only a segment of intracellular structural domains with no kinase activity, and Receptor-Like Kinases (RLKs), which usually consist of kinase, transmembrane, and ligand structural domain (Gui et al., 2017). *BAK1* and *SOBIR1* are two conserved RLKs with short leucine-rich repeat sequences in their extracellular domains. These domains act as coreceptors for various MAMP-induced immune responses (Burgh et al., 2019). The results showed that PeVn1-induced cell death was dependent on *BAK1* and *SOBIR1*, further confirming that PeVn1 is a MAMP. Nonetheless, further studies are needed to identify the receptors that can recognize PeVn1 (**Figure 7**).

The most widely utilized signaling pathways in plants are the SA and JA/ET (van Wees et al., 2000; Nomoto et al., 2021; Zhu et al., 2021). Some evidence suggests that depending on the specific pathogen, the SA, JA, and ET pathways can interact with each other and modulate plant defense responses (Koornneef and Pieterse, 2008). In the present study, PeVn1 significantly upregulated the SA and JA/ET genes in *N. benthamiana* (**Figure 5C**), indicating that PeVn1 induced the expression of genes related to SA synthesis in *N. benthamiana* (*NbPR1* and *NbPR2*). The significant upregulation of JA-responsive genes (*NbPR4*, *NbCOI*, and *NbPDF1*.2) and ET-responsive gene *NbERF1* suggest that PeVn1 induces JA gene expression and ET synthesis in tobacco. The process of ROS accumulation is regulated by a series of enzymes that function to detoxify ROS, including SOD, CAT, POD and glutathione reductase (Apel and Hirt, 2004). These results suggest the potential activation of multiple signaling pathways in PeVn1 triggered resistance, enhancing plant resistance to pathogens under PeVn1 treatment. It was found that PeVn1 induced activities of PAL, CAT, POD and SOD (**Figure 6**). To determine whether the PeVn1 mediated defense response of *N. benthamiana* is resistant to various phytopathogens, we selected *S. sclerotiorum*, *B. cinerea* and TMV-GFP. The results showed that the treatment of PeVn1 resulted in a significant reduction in lesion area and relative biomass. In addition, recombinant protein PeVn1 treatment also induced resistance in plants against a variety of pathogenic fungi and bacteria.

Overall, our study reports a novel protein elicitor PeVn1 from *V. nonalfalfae* HW that induces *N. benthamiana* HR and acts as a MAMP to induce plant immune responses, and PeVn1 induces *BAK1* and *SOBIR1* dependent cell death and activates defense related events in *N. benthamiana* and enhanced plant resistance to *S. sclerotiorum*, *B. cinerea*, TMV-GFP and *Pst* DC3000. These findings further elucidate the biological functions of PeVn1 and the molecular mechanisms underlying the regulation of plant defense mechanisms, thereby establishing a foundation for future research.

## 4 Materials and methods

### 4.1 Strain culture and plant growing conditions

*Verticillium nonalfalfae* HW was cultured in PDA medium at 25°C for 2 weeks, and then mycelium at the edge of the colony was picked and inoculated with the Czapek medium and cultured in the dark at 25°C for 14 days at 150 rpm, *S. sclerotiorum* and *Botrytis cinerea* were cultured in PDA medium at 25°C for 3 days, *Pseudomonas syringae* pv. *tomato* (Pst) DC3000 was cultured in KB medium at 28°C with 150 rpm shaking overnight, *E. coli* DH5α and BL21 (DE3) were incubated in LB medium at 37°C, 180 rpm on a shaker. *A. tumefaciens* GV3101 25 μg/mL rifampicin was incubated in LB medium containing *A. tumefaciens* GV3101 25 μg/mL rifampicin. 28°C, 220 rpm, 18 h.

*Nicotiana benthamiana* and tomato plants were cultivated at 25°C with a photoperiod of 16 h and an 8 h dark period. All strains and seeds were provided by the Plant Pathology Laboratory of the College of Horticulture and Plant Protection, Inner Mongolia Agricultural University. Tomato seeds were purchased from the Meng Miao Seed Company (Hohhot, China).

### 4.2 Extraction, isolation and identification of proteins secreted from strain HW

Strain HW was incubated for 7 days, before collecting the supernatant. The supernatant was then collected by centrifugation at 13,000 rpm for 30 min at 4°C. Subsequent to centrifugation at 13,000 rpm for 30 min at 4°C, the supernatant was filtered twice with a 0.45 μm filter (Millipore, Suzhou, China) until no organisms were present. Proteins were precipitated overnight at 4°C by the addition of a 75% ammonium sulfate solution. Following centrifugation, the precipitate was dissolved in 20 mM Tris-HCl and dialyzed for a period of two days. The dialyzed proteins were further purified using the gel filtration-preloaded columns of the AKTA protein purification system (Superdex 200 Increase 10/300 GL; Cytiva, MA, USA). The purified proteins were tested for cell death activity in *N. benthamiana* leaves, and the fractions with cell death activity were separated by sodium dodecyl sulfate polyacrylamide gel electrophoresis. A single band was cut and identified by liquid chromatography/mass spectrometry.

### 4.3 Purification and expression of PeVn1 in prokaryotic systems

The PeVn1 sequence, which does not contain a signal peptide, was inserted between the *Bam*HI and *Xho*I sites of the *pET* vector to construct the plasmid. **Supplementary Table 1** lists all the primers used. The plasmids were then expressed in *E. coli* BL21 (DE3), and the expression of the target proteins was induced by adding 1 mM IPTG to the induced culture at 16°C, 80 rpm for 12 h. The expression of the target proteins was then induced by adding 1 mM IPTG to the induced culture at 16°C, 80 rpm. After centrifugation, the organisms were collected, washed thrice in phosphate-buffered saline (PBS) (20 mM Na_2_HPO_4_, 300 mM NaCl, pH 8.0), and lysed by ultrasonication to obtain a crude extract of the recombinant protein. Purification of the filtrate was achieved through affinity chromatography, employing HisPur Ni-NTA agarose (Cytiva), and the concentration of PeVn1 was quantified by BCA protein assay. When the purified protein concentration exceeded 0.1 mg/mL, it was dialyzed with PBS for subsequent experiments.

### 4.4 Protein expression in *N. benthamiana*

For cell death assays, *A. tumefaciens* GV3101 constructed with *PVX-PeVn1-GFP*, *PVX-eGFP* or *PVX-Bax* in the potato virus X (PVX) vector was washed three times with 10 mM MgCl_2_ and diluted to an OD_600_ of 0.8. The corresponding infusate was injected into the dorsal part of the leaf using a 1 mL syringe. To clarify the exact location of PeVn1 function, *pCAMBIA1300-PeVn1-GFP* and *pCAMBIA1300-PeVn1^ΔSP^-GFP* were transformed into *A. tumefaciens* and the suspension with OD_600_ = 0.8 was injected into 4 week *N. benthamiana* leaf blades in a glass greenhouse at 22°C. At 48 hpi, the suspension was incubated in a glass greenhouse for 2 h and washed thrice with 10 mM MgCl_2_. greenhouse at 22°C. At 48 h post infection (hpi), the leaf epidermis was treated and observed under a confocal microscope.

### 4.5 Bioinformatic analysis of PeVn1

To conduct protein structure analysis of PeVn1, its signal peptide was predicted using the SignaIP website (http://www.cbs.dtu.dk/services/SignalP/), and the protein transmembrane helix was predicted using the TMHMM website (http://www.cbs.dtu.dk/services/TMHMM/). The primary protein structural information, such as molecular weight and isoelectric point of PeVn1, was analyzed using ProParam (http://web.expasy.org/cgi-bin/protparam/protparam) (Kumar et al., 2018). The tertiary structure was predicted using SWISS-MODEL software (https://www.swissmodel.expasy.org/) (Waterhouse et al., 2018). The amino acid sequences were obtained from the UniProt database (https://www.uniprot.org/). Homologous sequences from the NCBI database were compared using BLASTp and 15 sequences with high similarity were selected. Sequence conservation was analyzed using DNAMAN software. Phylogenetic dendrograms were constructed using MEGA 6.0 and the neighbor-joining method. PeVn1 data were submitted to the NCBI database under the GenBank number XP 028497723.1.

### 4.6 Transient expression of proteins in yeast

The signal peptide sequence was cloned into the *pSUC2* vector, which carries the sucrose invertase gene SUC2 without the initiation ATG codon and the signal peptide sequence and was transformed into yeast YTK12 (Jacobs et al., 1997). The transformant strains were then screened on CMD−W plates (0.67% yeast nitrogen base, 2% sucrose, 0.1% glucose, 2% agar, 0.075% tryptophan dropout supplement) and selective YPRAA plates (1% yeast extract, 2% peptone, 2% raffinose, 2 μg/ml antimycin A, 2% agar). YTK12 strains with empty *pSUC* vector or *pSUC2-Avr1b* were used as negative and positive controls, respectively. The enzymatic activity was tested by reducing 2,3,5-triphenyl tetrazolium chloride to red 1,3,5-triphenyl formazan.

### 4.7 Determination of the cell death response of recombinant proteins to different crops

To test the necrotic activity of the recombinant protein against different plants, 5 μm of the recombinant protein was infiltrated into the leaves of *N. benthamiana* and also into the leaves of potato (*Solanum tuberosum* L.), oat (*Avena* L.), and tomato (*Solum lycopicum*). Photographs were taken three days after infiltration. The experiment was repeated with at least three biological replicates. *N. benthamiana* leaves were immersed in 1 mg/mL trypan blue dye solution and boiled for 2 min. After standing to return to room temperature, the leaves were rinsed 3-4 times with sterile water and decolorized by adding 2.5 g/mL chloral hydrate.

### 4.8 Physicochemical properties and induction of early plant responses by PeVn1

Determination of the lowest excitation concentration of recombinant protein PeVn1, the recombinant protein PeVn1 was injected into *N. benthamiana* leaves at concentrations of 1, 5, 10, 20 and 50 μM. The thermal stability of the recombinant protein PeVn1 was determined. The recombinant protein PeVn1 was injected into *N. benthamiana* at an appropriate concentration later, the leaves were incubated at 100°C for 15 min. The PeVn1 pure protein of appropriate concentration was injected into *N. benthamiana* to observe its immune response. Leaves at 24 h posttreatment were cut into small pieces, 1 × 1 cm. H_2_O_2_ was detected by DAB staining and callose was detected by aniline blue staining as described previously (Niu et al., 2011). ROS burst was identified via light microscopy, while callus deposition was observed using a fluorescence microscope (Nikon, Japan).

### 4.9 MAPK assay

Leaves of *N. benthamiana* were treated with 5 μM PeVn1, and samples were taken at 0, 5, 20, and 30 min to extract total plant proteins with RIPA buffer (50 mmol/L Tris pH7.5, 150 mmol/L NaCl, 1% Triton X-100, 1% deoxycholate, 1% SDS, 0.5 mmol/L EDTA, 1×PMSF, 1×protease inhibitor cocktail) to extract total plant protein. Western blot was performed using pERK1/2 antibody.

### 4.10 Bioassay for PeVn1-induced disease resistance in plants

The leaves were treated with 5 μM PeVn1 and PBS (Control) for 24 h. After 24 h, cultured *S. sclerotiorum* and *B. cinerea* were inoculated onto the leaves and incubated in the greenhouse at 25°C for 24 to 72 h to observe the onset of disease. Photographs were taken and the lesions’ size was calculated using ImageJ software at 48 hpi. An equal volume of 5 μM PeVn1 and PBS was sprayed on 5-6 week old tomato leaves, which were incubated at 24°C under humid conditions for 3 days. After centrifugation of the cultured *Pst* DC3000 sap, the bacterial body was collected and suspended using 10 mM MgCl_2_ with OD_600_ = 0.1, before uniformly spraying on the leaves. After 3 days, three leaves were taken from each treatment and ground into a powder. The powder was diluted to 10^-6^ concentration using sterile water, coated on King’s B medium containing 50 μg/mL rifampicin at 37°C, and the number of colonies was determined after 2 days. The number of colonies was determined by the *Pst* DC3000 method. The experiment was repeated at least thrice. *N. benthamiana* leaves were treated with 5 μM PeVn1 and PBS (Control). After 24 h, the leaves were inoculated with *TMV-GFP*. For *TMV-GFP* inoculum preparation, leaves previously infected with *TMV-GFP* were ground, homogenized in PBS buffer, and centrifuged at 3500 g for 10 min. The supernatant was collected and inoculated onto leaves using the rubbing method. After inoculation and incubation for 4 days, the number of spots was counted under a UV lamp. Three replicates were performed for each treatment group. Viral suppression was calculated as follows: suppression (%) = (Nc-NT)/Nc × 100, where Nc and NT represent the number of lesions on the control and PeVn1-treated leaves, respectively.

### 4.11 Determination of defense-related enzyme activities after PeVn1 treatment of tobacco

The activities of SOD, POD, CAT and PAL were determined in PeVn1 (5 μM) treated *N. benthamiana* leaves at different intervals (0-8 days). Samples of treated *N. benthamiana* leaves (0.1 g) were collected at different intervals (0-8 days) and immediately frozen in liquid nitrogen. At least three samples were used to determine enzyme activity. Frozen tissue samples were treated as previously described (Zhang et al., 2015). First, the samples with 3 mL of PBS (20 mM Na_2_HPO_4_, 300 mM NaCl, pH 8.0) with 1.33 mM ethylenediaminetetraacetic acid and 1% (w/v) polyvinyl-pyrrolidone were ground at 4°C. The homogenate was centrifuged at 8000 g for 20 min, and then the obtained extract was 150 and subjected to enzyme assay for SOD, POD, CAT, and PAL. The activities of these enzymes were determined using assay kits (Sinobestbio, Shanghai, China) according to the manufacturer’s instructions.

### 4.12 RNA extraction and RT-qPCR analysis

Total RNA was extracted from *N. benthamiana* leaf blades using a Plant RNA Extraction Kit (TAKARA). cDNA was reverse transcribed using PrimeScript™ RT Master Mix (TAKARA). Quantitative PCR was performed using the LightCycler 96 System (Roche) and TB Green^®^ Premix Ex Taq™ II (TAKARA) to determine gene expression. All experiments consisted of three biological and three technical replicates, and the *EF1α* gene in *N. benthamiana* was used as a reference control for normalization. The relative expression of each gene was calculated using the 2^-ΔΔCt^ comparison method (Livak and Schmittgen, 2001). **Supplementary Table 1** lists all the primers used for RT-qPCR.

### 4.13 VIGS in *N. benthamiana*

To silence *NbBAK1* and *NbSOBIR1*, the target genes were cloned into the *pTRV2* vector, with *pTRV2:PDS* and *pTRV2:GFP* used as positive and negative controls, respectively. The vectors were *de novo* synthesized in *A. tumefaciens* GV3101. Bacterial fluids Infiltrated into *N. benthamiana* leaf blades at an OD_600_ of 0.8 After a 2 weeks period, the silencing efficiency of *BAK1* and *SOBIR1* was determined by RT-qPCR at the location of the treated leaves. The albino phenotype due to PDS gene silencing was used as a reference.

### 4.14 Statistical analysis

All the data presented are the mean values of three biological repetitions and were statistically analyzed via one-way ANOVA and Tukey’s test (p < 0.05) using GraphPad Prism 8.0 software.

## Data Availability

The datasets presented in this study can be found in online repositories. The names of the repository/repositories and accession number(s) can be found below: https://www.ncbi.nlm.nih.gov/genbank/, XP 028497723.1.
