# Supplementary material for: A protein elicitor PeVn1 from Verticillium nonalfalfae HW recognized as a MAMP triggers plant immunity response
Source: Front Plant Sci. 2024 Oct 10;15:1468437. doi: 10.3389/fpls.2024.1468437 (PMC11499194; doi:10.3389/fpls.2024.1468437)
Supplement: Supplementary file 1 [file DataSheet1.docx]

Supplementary Material

# Supplementary Figures and Table

## Supplementary Figures

## Supplementary Tables

# Extended data Figures

## 1.1 Supplementary Figures


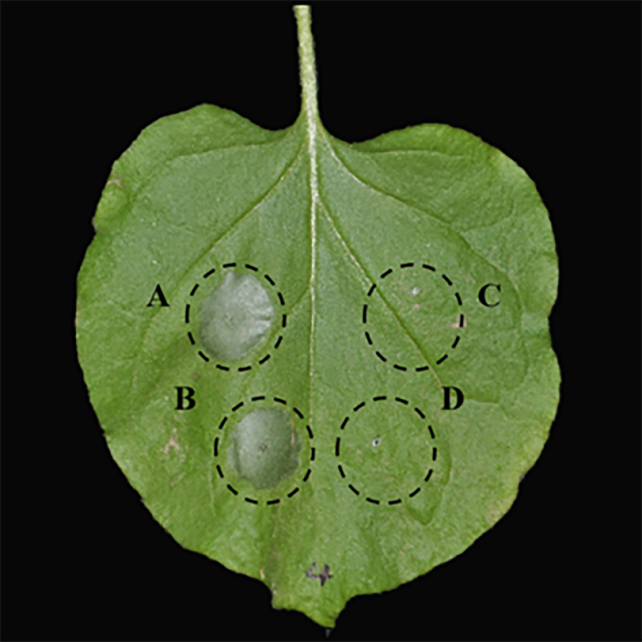


**Supplementary Figure S1.** The cell death activity of strains HW crude exoprotein. **(A)** Fermentation supernatant of *V. nonalfalfae* HW. **(B)** The crude exoprotein was separated using ammonium sulfate solution. **(C,D)** 20mM Tris-HCl protein buffer. was used as a control.

**
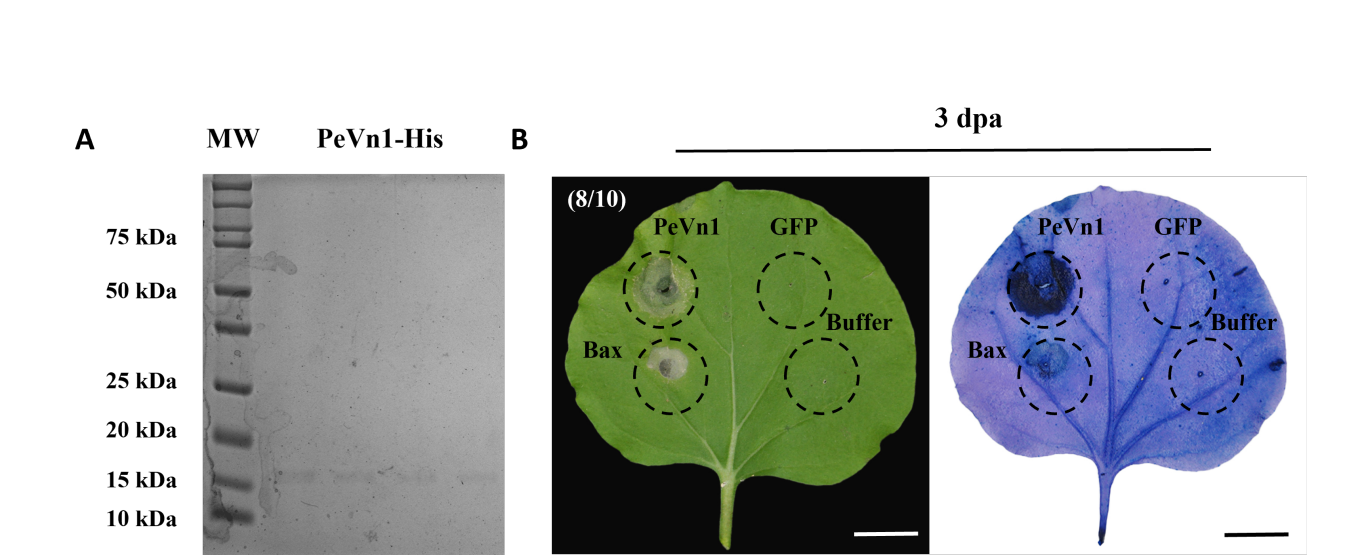
**

**Supplementary Figure 2.** Purification of recombinant protein PeVn1 and necrotic response of plant cells. **(A)** Purification of recombinant PeVn1 was analyzed by SDS-PAGE. PeVn1: Purified His tagged PeVn1. **(B)** PeVn1 induced cell death in tobacco. Leaves of *N. benthamiana* were infiltrated with recombinant PeVn1 and His tag, respectively. Bax was used a positive control. Green fluorescent protein (GFP) and buffer were used as a negative control. Bars are 1 cm.


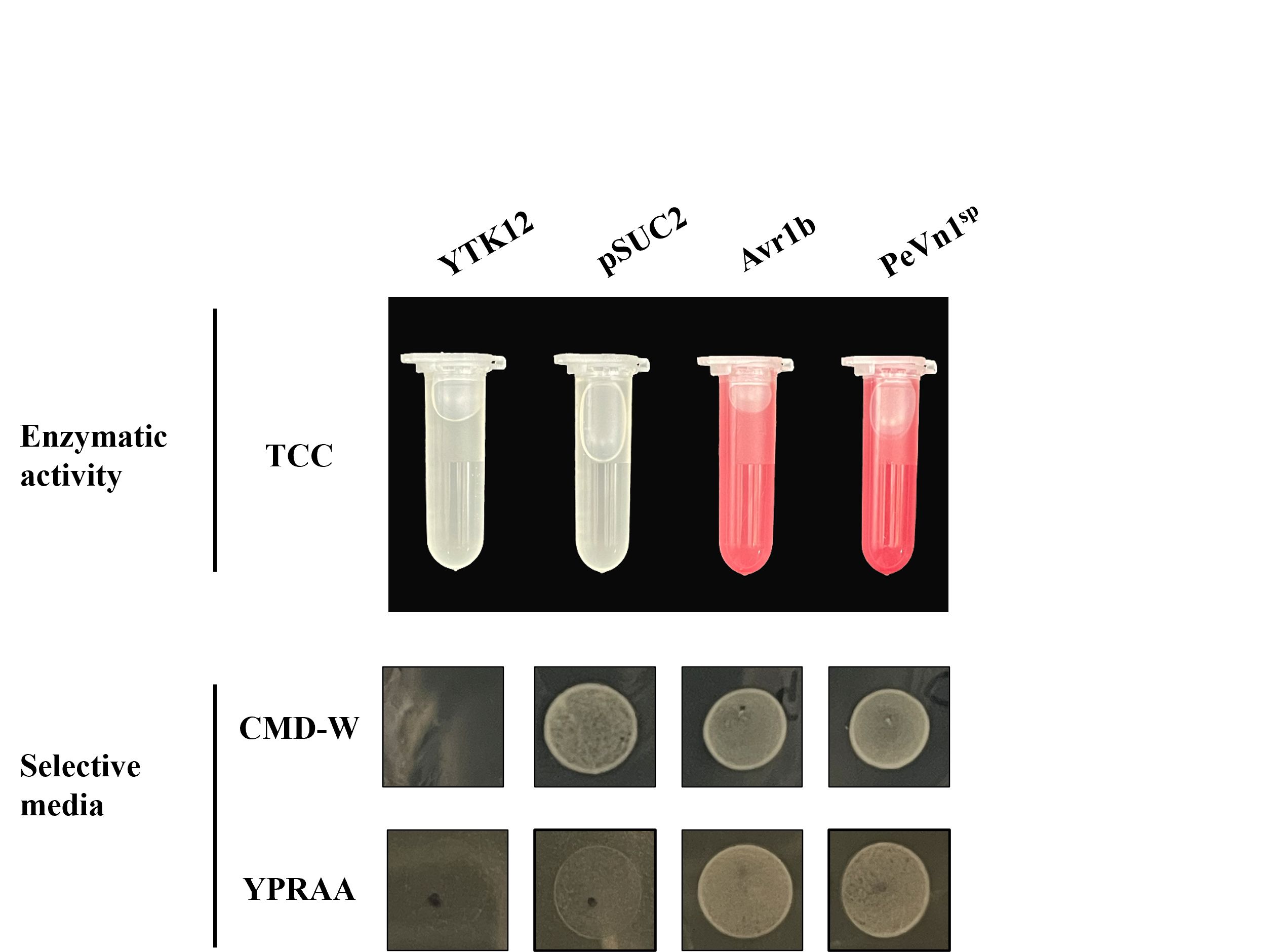


**Supplementary Figure 3.** The secretion of signal peptide (SP) from PeVn1. The yeast SUC2 mutant YTK12 and its transformants expressing the empty vector *pSUC2* or vectors with the signal peptide from PeVn1 and Avr1 (positive control) were assayed for growth on CMD-W or YPRAA plates and invertase activity in TTC medium.

## 1.2 Supplementary Tables

**Supplementary Table 1.** Primers used for qRT-PCR in this study

| **Primer name** | **Sequence (5′to 3′)** |
| --- | --- |
| qRT-*NbEF1α*-F | CATCGAGAAGTTCGAGAAGG |
| qRT-*NbEF1α*-R | TACTTGAAGGAACCCTTACC |
| qRT-*NbPR1*-F | CCGCCTTCCCTCAACTCAAC |
| qRT-*NbPR1*-R | GCACAACCAAGACGTACTGAG |
| qRT-*NbPR2*-F | AGGTGTTTGCTATGGAATGC |
| qRT-*NbPR2*-R | TCTGTACCCACCATCTTGC |
| qRT-*NbPR4*-F | GGCCAAGATTCCTGTGGTAGAT |
| qRT-*NbPR4*-R | CACTGTTGTTTGAGTTCCTGTTCCT |
| qRT-*COI1*-F | AACTGGTCGGGATCTCTTGG |
| qRT-*COI1*-R | TAGGCAAGTATATGGGCGGG |
| qRT-*NbERF1*-F | GCTCTTAACGTCGGATGGTC |
| qRT-*NbERF1*-R | AGCCAAACCCTAGCTCCATT |
| qRT-*NbPDF1.2*-F | ATCTGTCTGGGGAAATGGCA |
| qRT-*NbPDF1.2*-R | CATGGTCCCTTGAAACGGTG |
| qRT-*NbCYP71D20*-F | AAGGTCCACCGCACCATGTCCTTAGAG |
| qRT-*NbCYP71D20*-R | AAGAATTCCTTGCCCCTTGAGTACTTGC |
| qRT-*NbWRKY8*-F | AACAATGGTGCCAATAATGC |
| qRT-*NbWRKY8*-R | TGCATATCCTGAGAAACCATT |

## 2 Extended data Figures


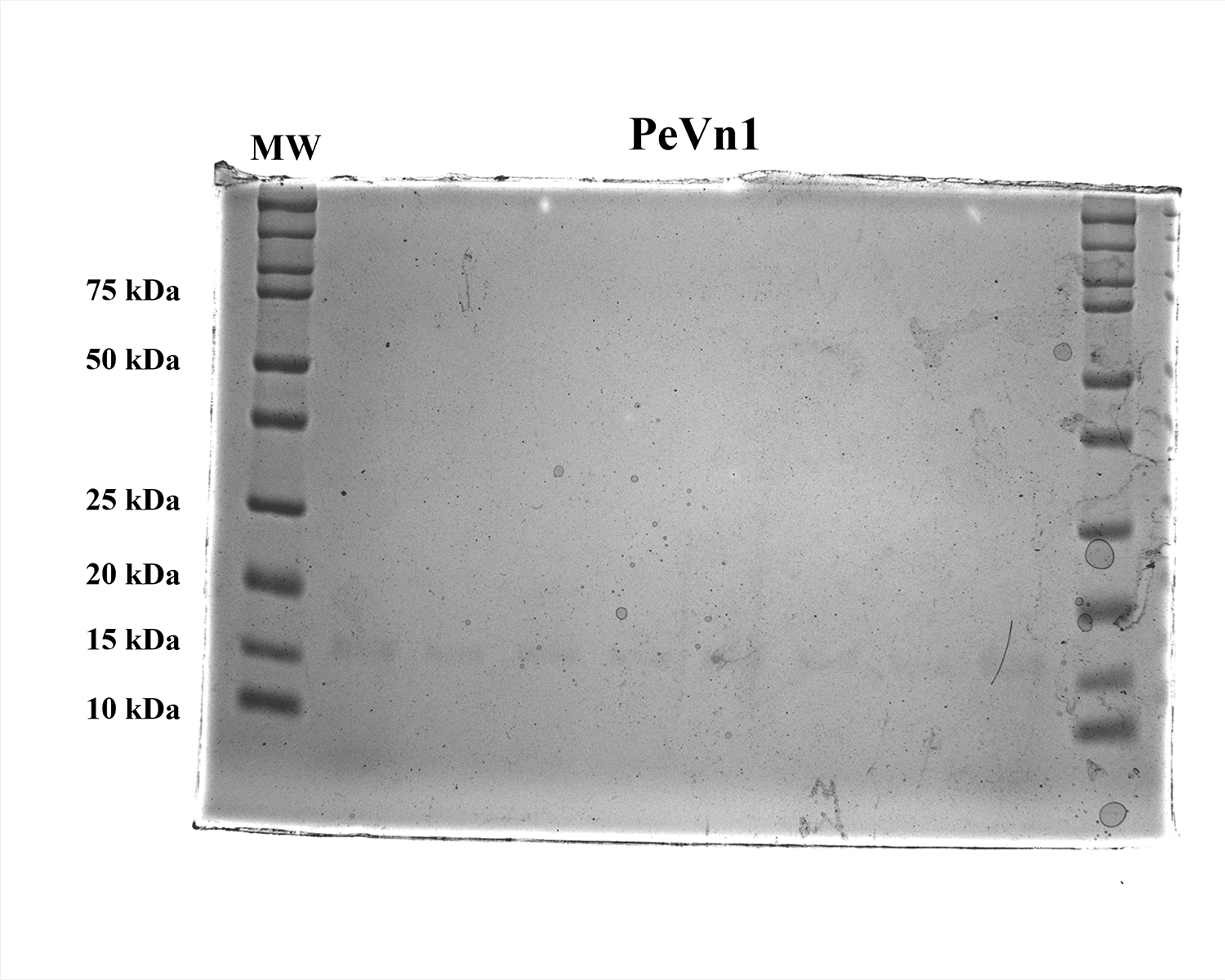
**Figure 1**

**Figure 5c**

**
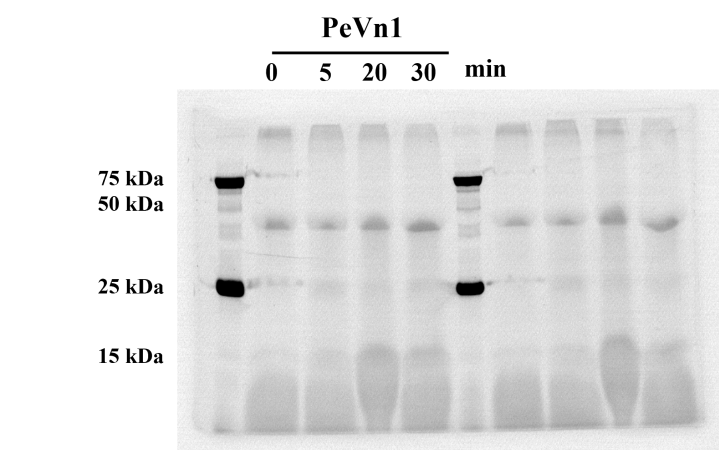

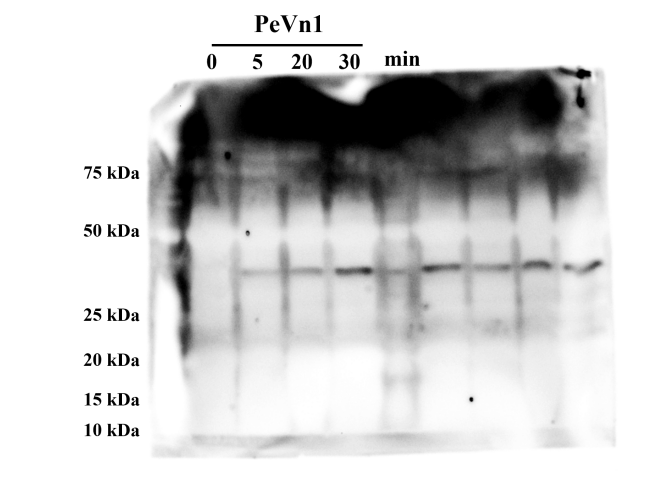
**

**
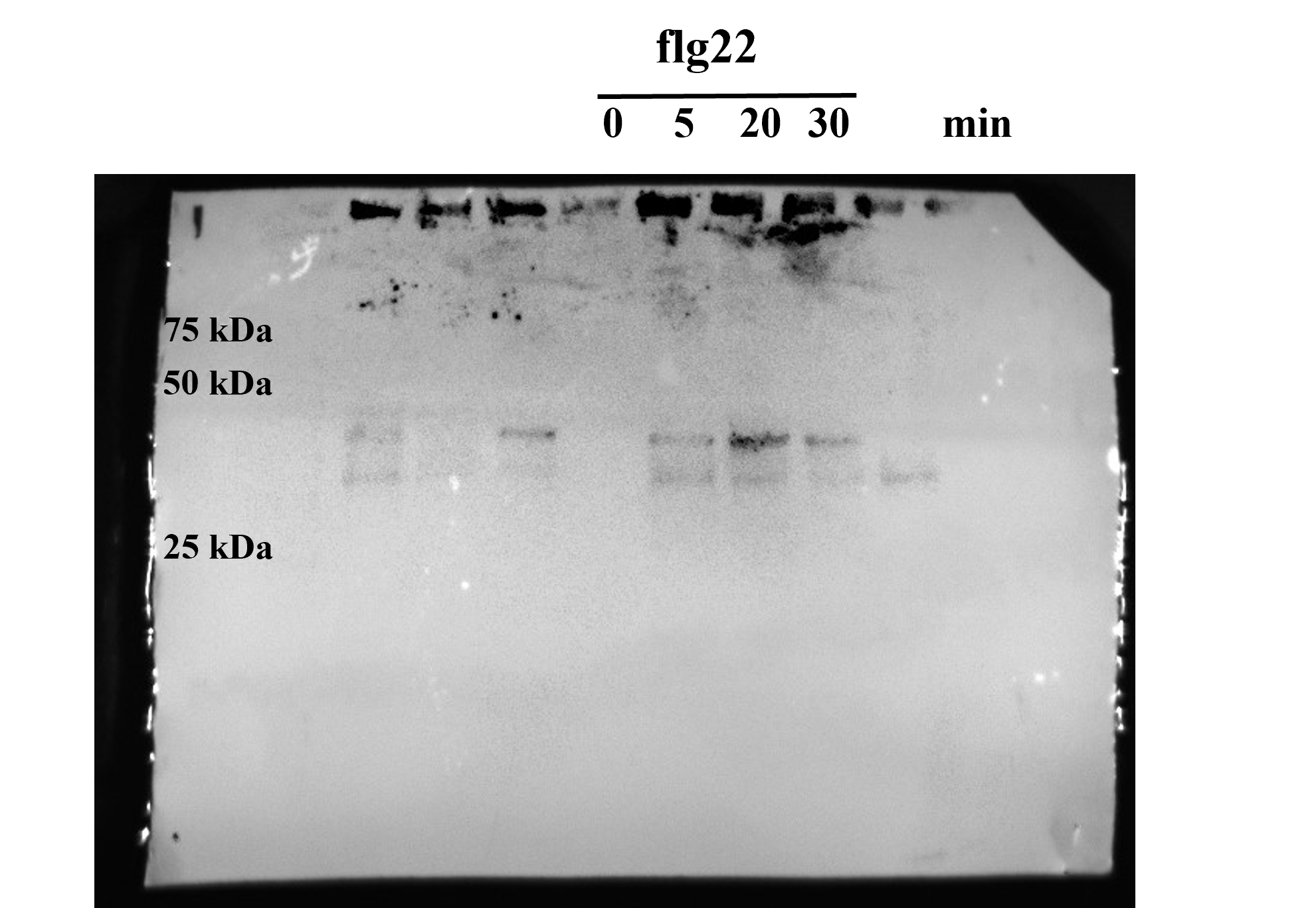

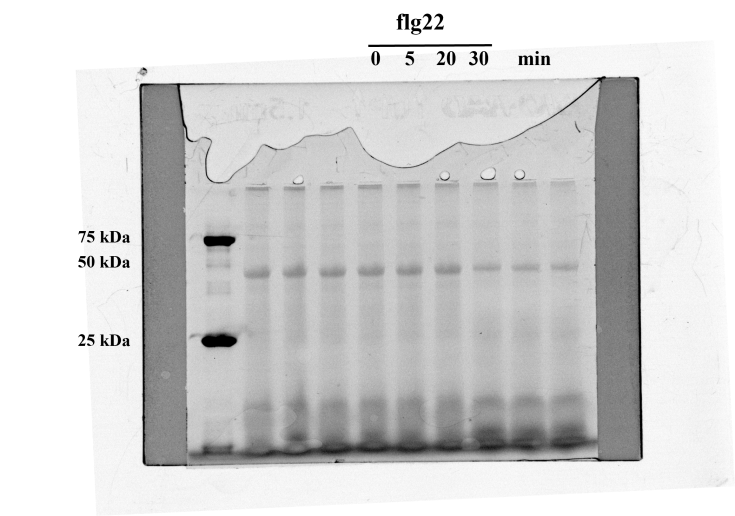
**

**Figure 7c**

**
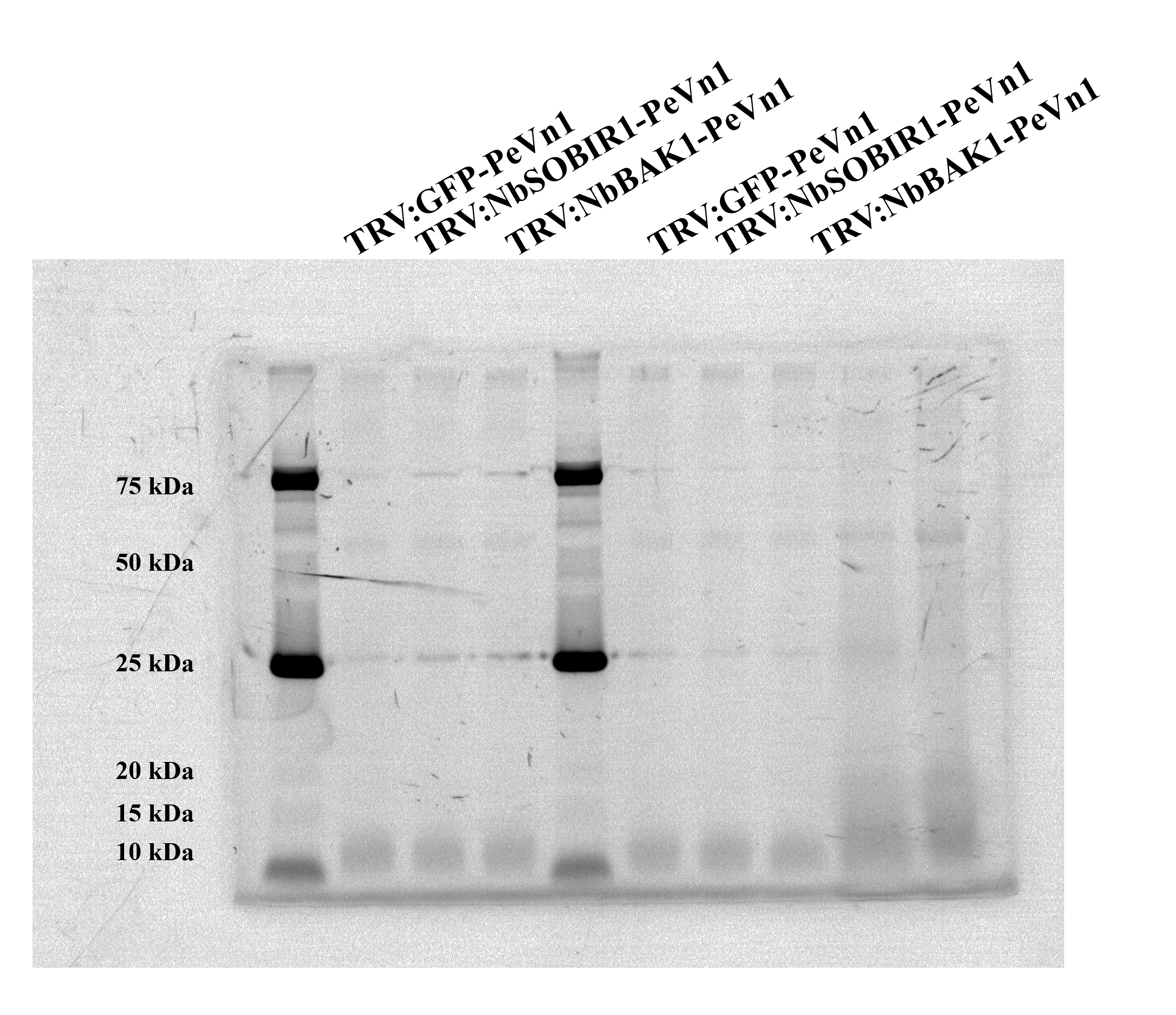
**

**
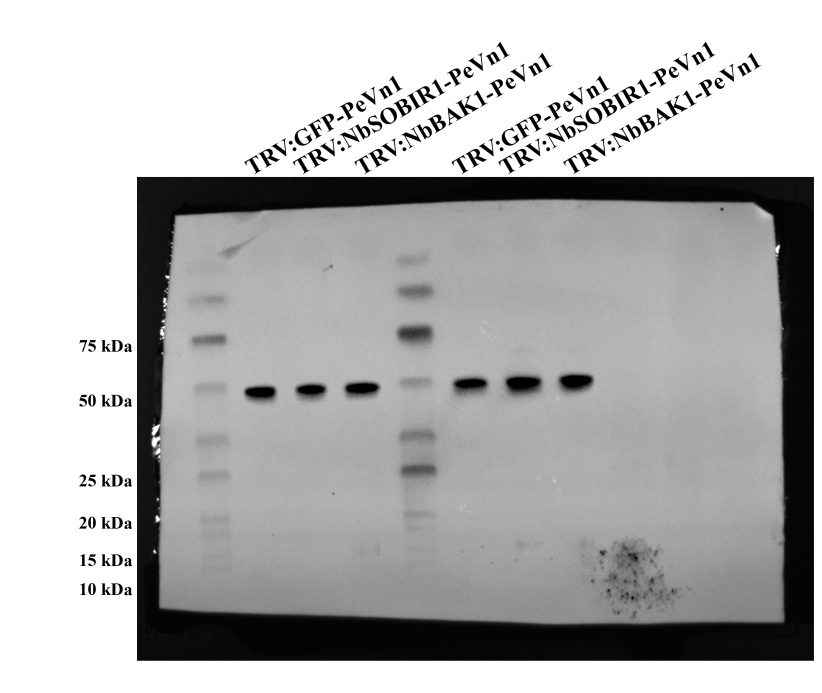
**

**
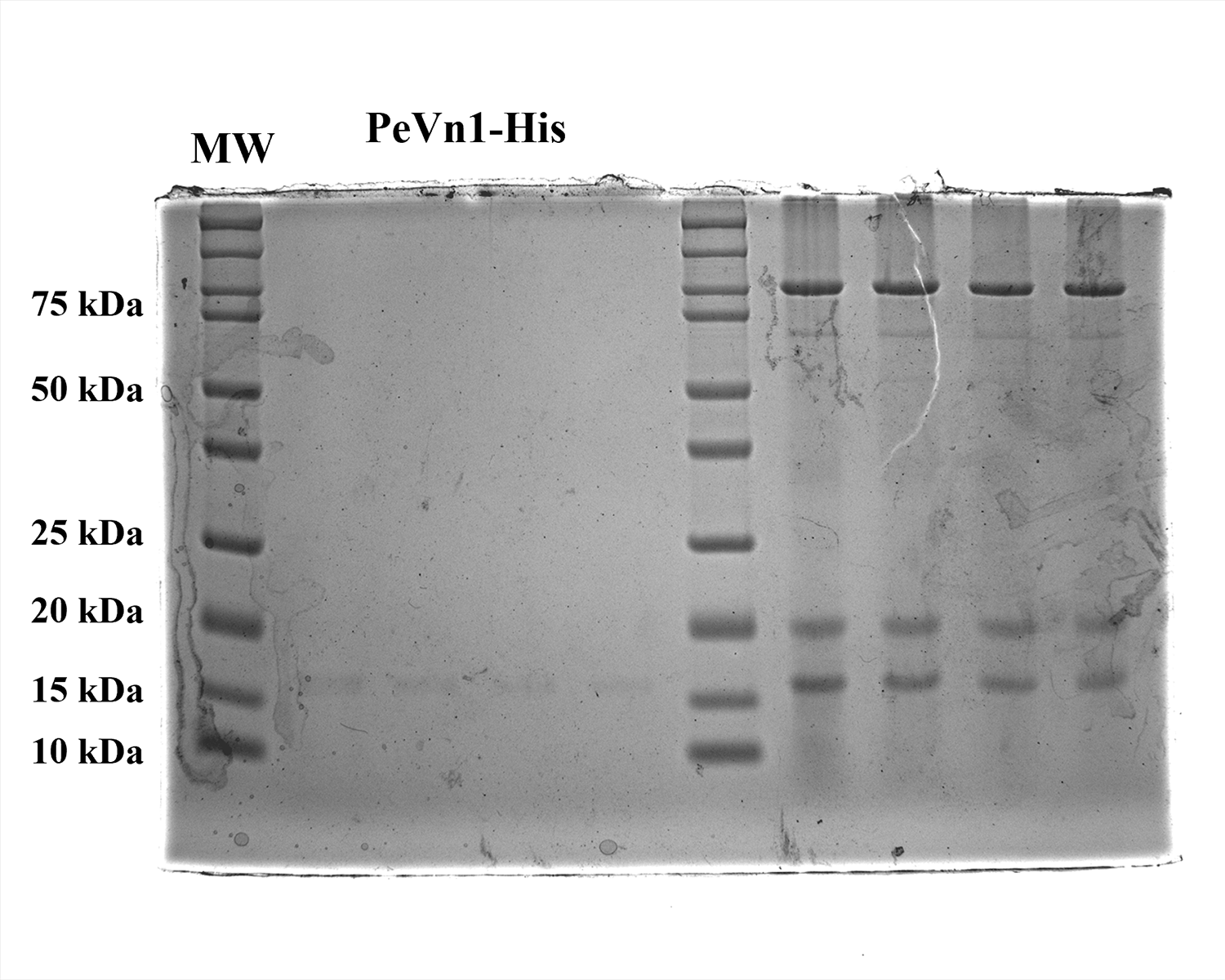
Supplementary Figure S2**
